# Nucleo-cytoplasmic shuttling of murine RBPJ by Hairless protein matches that of Su(H) protein in the model system *Drosophila melanogaster*

**DOI:** 10.1186/s41065-021-00175-z

**Published:** 2021-03-28

**Authors:** Dorina B. Wolf, Dieter Maier, Anja C. Nagel

**Affiliations:** Department of General Genetics (190g), University of Hohenheim, Garbenstr. 30, 70599 Stuttgart, Germany

**Keywords:** Notch signal transduction, Hairless, CSL, CBF1, RPBJ, Su(H), Protein availability, Nucleo-cytoplasmic transport, Transcription repression, *Drosophila*

## Abstract

**Abstract:**

CSL transcription factors are central to signal transduction in the highly conserved Notch signaling pathway. CSL acts as a molecular switch: depending on the cofactors recruited, CSL induces either activation or repression of Notch target genes. Unexpectedly, CSL depends on its cofactors for nuclear entry, despite its role as gene regulator. In *Drosophila*, the CSL homologue Suppressor of Hairless (Su(H)), recruits Hairless (H) for repressor complex assembly, and eventually for nuclear import. We recently found that Su(H) is subjected to a dynamic nucleo-cytoplasmic shuttling, thereby strictly following H subcellular distribution. Hence, regulation of nuclear availability of Su(H) by H may represent a new layer of control of Notch signaling activity. Here we extended this work on the murine CSL homologue RBPJ. Using a ‘murinized’ fly model bearing *RBPJ*^*wt*^ in place of *Su(H)* at the endogenous locus we demonstrate that RBPJ protein likewise follows H subcellular distribution. For example, overexpression of a H^*NLS3^ protein variant defective of nuclear import resulted in a cytosolic localization of RBPJ protein, whereas the overexpression of a H^*NES^ protein variant defective in the nuclear export signal caused the accumulation of RBPJ protein in the nucleus. Evidently, RBPJ is exported from the nucleus as well. Overall these data demonstrate that in our fly model, RBPJ is subjected to H-mediated nucleo-cytoplasmic shuttling as is Su(H). These data raise the possibility that nuclear availability of mammalian CSL proteins is likewise restricted by cofactors, and may hence present a more general mode of regulating Notch signaling activity.

**Graphical abstract:**

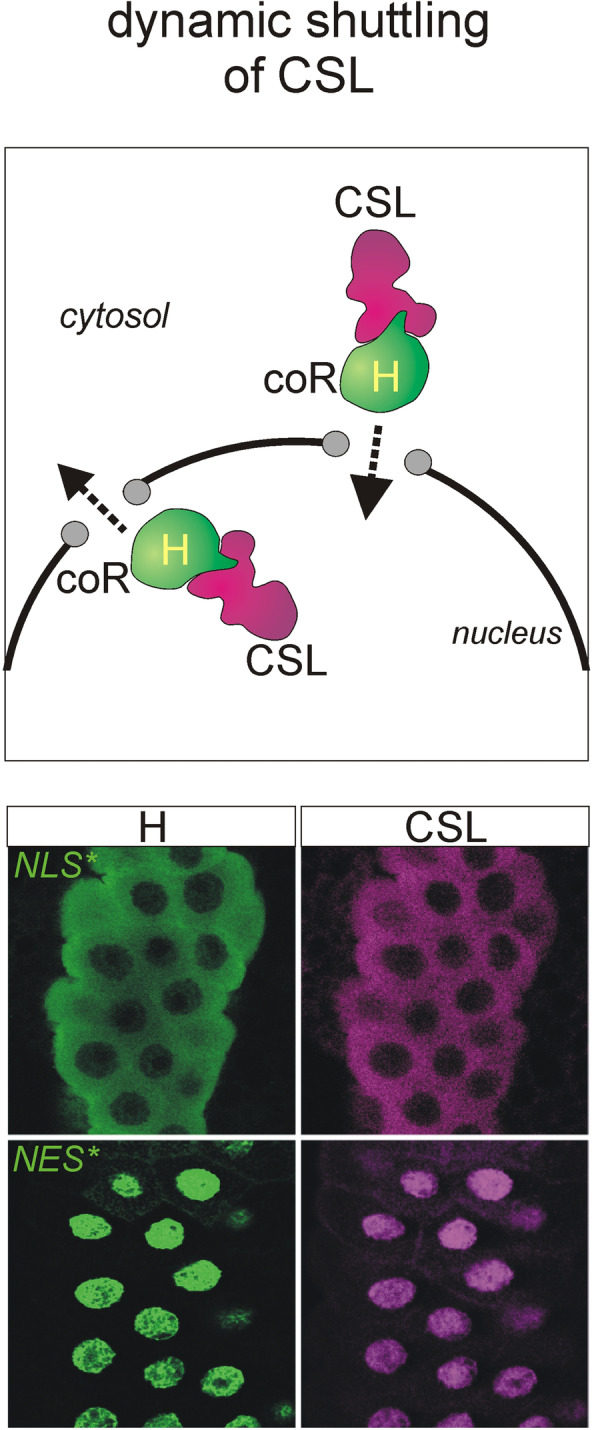

## Background

Development as well as tissue homeostasis of higher eumetazoa depends on inter-cellular communication mediated by the Notch signaling pathway. Accordingly, the Notch signaling pathway is highly conserved in the evolution of invertebrates and vertebrates alike [1–3]. Upon binding of one of its ligands, the Notch receptor undergoes cleavage releasing the Notch intracellular domain NICD. Together with several cofactors, NICD assembles a transcriptional activator complex switching gene expression, and eventually cell fate, in the signal-receiving cell [3–7]. Pivotal to Notch target gene regulation is the DNA binding protein CSL; CSL is an acronym for mammalian CBF1/ RBPJ, for *Drosophila* Su(H) and for *Caenorhabditis* Lag1. Crystal structure analyses of the trimeric activator complex revealed that NICD contacts CSL with its RAM-domain and ankyrin repeats, allowing recruitment of the coactivator Mam [8]. In the absence of signal, CSL engages in Notch target gene inhibition by forming a repressor complex on Notch target gene promoters [9, 10]. Several corepressors have been identified in mammals, which compete with NICD for the RAM-binding site within the beta-trefoil domain of CSL [9–12]. The major antagonist of the Notch signaling pathway in *Drosophila* is named Hairless (H) [13]. Contrary to most of the mammalian CSL corepressors, H contacts the C-terminal domain of the fly CSL homologue named Suppressor of Hairless (Su(H)) [14–16]. By recruiting two general corepressors, Groucho and C-terminal binding protein, the Su(H)-H repressor complex eventually silences Notch target genes [13, 17–19]. With SHARP (also named MINT), a functional homologue of H has been identified in vertebrates [9, 11, 20, 21]. SHARP binds CSL in a bipartite manner, i.e. both within the beta-trefoil domain and the C-terminal domain, resembling the interaction of mammalian corepressors as well as of H with CSL [22].

Unexpected for a transcription factor, CSL apparently relies on its cofactors for nuclear entry. For example, mutations of CBF1/RBPJ in the beta-trefoil domain affecting both, the binding of NICD as well as of corepressors, prevented nuclear entry and Notch target gene activation [23]. Similarly, Su(H) nuclear entry depended on NICD in a *Drosophila* cell culture system; hence it may not enter the nuclear compartment on its own [24, 25]. Moreover, tissue-specific overexpression of H protein caused Su(H) nuclear accumulation, whereas Su(H) protein levels appeared reduced in the absence of H protein [26–28]. In fact, it was demonstrated that Su(H) protein stability depends on formation of transcription complexes together with H and NICD, respectively [28].

## Subcellular localization of Hairless and suppressor of Hairless protein

We recently addressed the subcellular localization of H and Su(H) proteins in *Drosophila* tissue and showed that Su(H) protein strictly follows the subcellular localization of H [29]. H protein contains three potential nuclear localization signals NLS1–3, with NLS3 being the most effectual. Accordingly, H^*NLS3^ mutant protein defective in NLS3 accumulated within the cytosol. In addition, a nuclear export signal NES, juxtaposed to NLS3, proved relevant for the export of H protein from the nuclear compartment. Mutation of the NES resulted in nuclear retention of H^*NES^ protein in larval tissues. Endogenous Su(H) protein co-localized with H protein, i.e. it was cytosolic when the H^*NLS3^ mutant was overexpressed and nuclear in cells expressing H^*NES^ [29]. A double mutant H^*NLS3*NES^ had an intermediate effect, and either protein distribution resembled the wild type situation, demonstrating the importance of the NES in H and Su(H) export. Overall our data implied, that H mediated shuttling of Su(H) between the nucleo-cytosolic compartments provided a means of regulating Notch activity by restricting nuclear availability of Su(H). Here we asked, whether mammalian CSL protein might be subjected to a similar mode of regulation. The fact that nuclear import of CBF1/RBPJ is dependent on its cofactors as well makes this hypothesis very likely. Moreover, in yeast two-hybrid assays murine CBF1/RBPJ was shown capable of binding H with its C-terminal domain similar to Su(H) [14, 15, 30].

## Murine RBPJ protein follows the subcellular distribution of Hairless protein in the fly

**Fig. 1:**
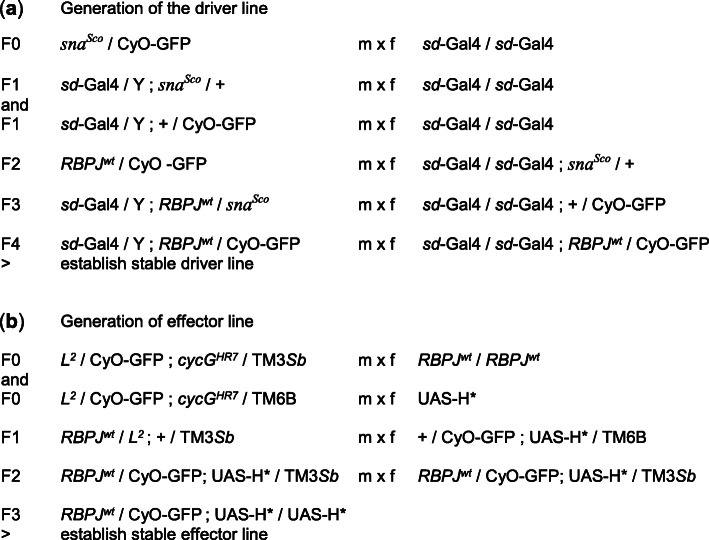
Crossing scheme. Crossing scheme for establishing (**a**) the driver line *sd*-Gal4; *RBPJ*^*wt*^ / CyO-GFP and (**b**) the effector lines *RBPJ*^*wt*^ / CyO-GFP; UAS-H* (representing the four different *H* alleles, UAS-*H*^*cwt*^, UAS-H^*NLS3^, UAS-H^*NES^ and UAS-H^*NLS3*NES^, respectively). Direction of the cross is indicated with males (m), and virgin females (f). Note that *sd*-Gal4 is X-linked. Crosses of driver and effector lines result in the desired offspring, i.e. third instar larvae homozygous for *RBPJ*^*wt*^ that can be selected for the absence of the GFP marker for subsequent analysis of salivary glands

To address the potential role for H on nucleo-cytoplasmic shuttling of mammalian CSL, we made use of a ‘murinized’ fly model which we recently established [30]. In these flies, the endogenous *Su(H)* locus has been replaced by the murine CSL orthologue RBPJ using genome engineering. Interestingly, *RBPJ*^*wt*^ flies are viable with subtle phenotypes, demonstrating that the murine CSL orthologue can replace the majority of Su(H) activities during fly development [30]. The *RBPJ*^*wt*^ fly model allowed us to test, whether RBPJ protein is subjected to H-mediated nuclear localization like its fly homologue Su(H), i.e. nuclear import – as expected by a likewise nuclear import of CBF1/RBPJ by corepressors – as well as nuclear export, as uncovered for Su(H) in *Drosophila*. We applied the Gal4-UAS system [31] for a tissue specific overexpression of H* variants mutant in a nuclear translocation signal, as this setting allows following the distribution of endogenous CSL protein within larval tissue [29]. For the overexpression, we used *sd*-Gal4 [32] driving UAS-H* transgene expression in the larval salivary glands, where subcellular protein localization can be easily visualized in the cytoplasm and nuclei of the giant cells [29]. To this end, we first combined the *RBPJ*^*wt*^ bearing 2nd chromosome with the *sd*-Gal4 line and the UAS-H* transgenes, respectively, to generate driver and effector lines in the *RBPJ*^*wt*^ genetic background (Fig. 1). Four *RBPJ*^*wt*^-bearing effector lines were established: UAS-*H*^*cwt*^ as control, UAS-H^*NLS3^ defective for nuclear import, UAS-H^*NES^ defective for nuclear export, and UAS-H^*NLS3*NES^ affecting both, import- and export signal [29].

**Fig. 2:**
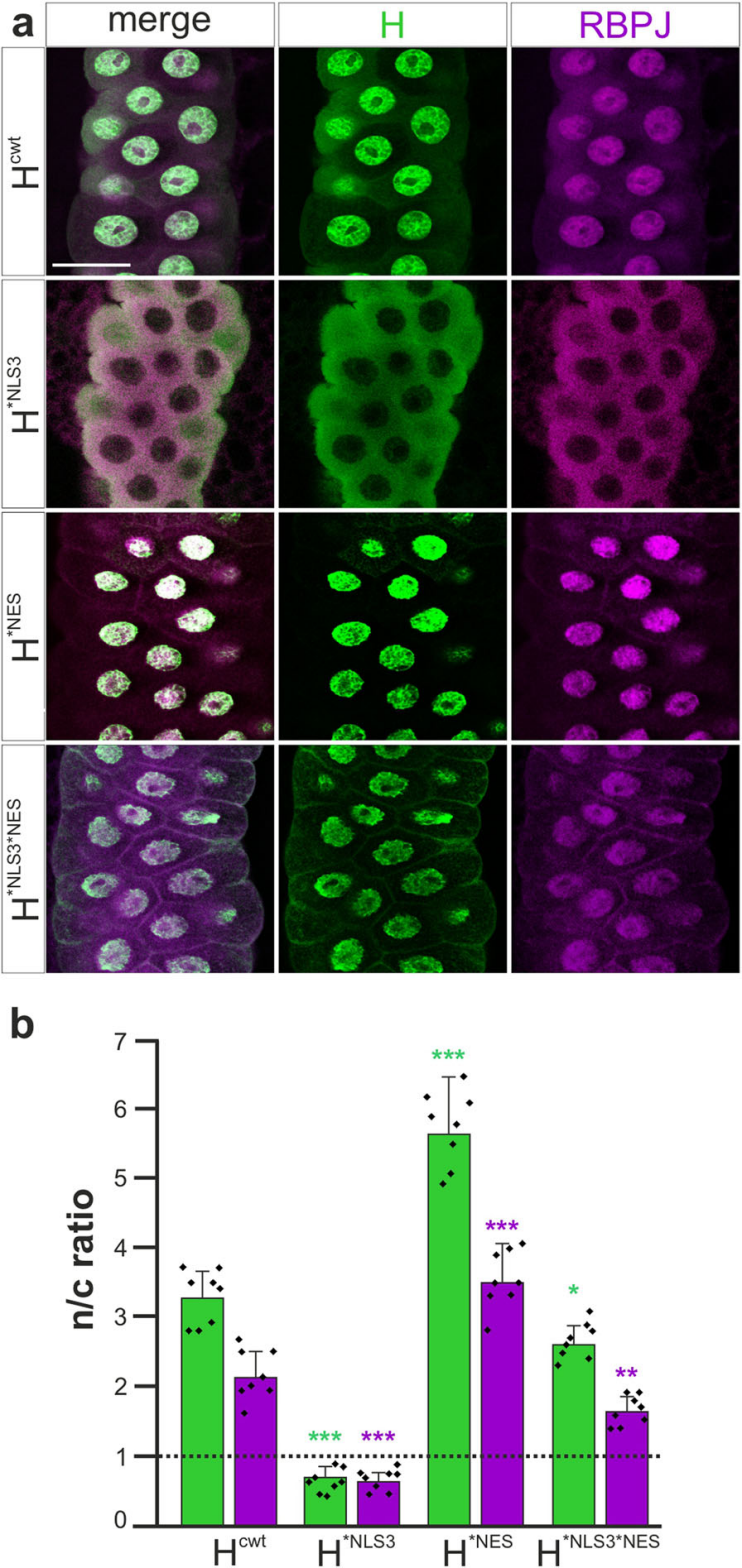
Subcellular co-localization of RBPJ and H proteins. **a** Enlargements of salivary glands derived from homozygous *RBPJ*^*wt*^ larvae overexpressing the indicated H* protein isoform. Subcellular distribution of H protein is shown in green and of RBPJ protein in magenta; the left panel shows the merge. Size bar represents 50 μm in all panels. The following genotypes are depicted: *sd*-Gal4/+; *RBPJ*^*wt*^ / *RBPJ*^*wt*^; UAS-*H*^*cwt*^/+, *sd*-Gal4/+; *RBPJ*^*wt*^ / *RBPJ*^*wt*^; UAS-H^*NLS3^/+, *sd*-Gal4/+; *RBPJ*^*wt*^ / *RBPJ*^*wt*^; UAS-H^*NES^/+, *sd*-Gal4/+; *RBPJ*^*wt*^ / *RBPJ*^*wt*^; UAS-H^*NLS3*NES^/+. **b** Nuclear to cytoplasmic (n/c) ratio is shown for H protein (green bars) and Su(H) protein (magenta bars), respectively, determined from 8 specimen each indicated as squares. Sample mean and standard deviation is indicated. The dotted line represents equal distribution in both compartments (i.e. nuclear equals cytoplasmic grey value). H^cwt^ is primarily nuclear, and H^*NES^ even more enriched in nuclei. In contrast, H^*NLS3^ is located in the cytosol, whereas H^*NLS3*NES^ is detected in the nuclear compartment as well. Note that RBPJ^wt^ strictly follows H* subcellular protein distribution. Statistical analysis was performed using ANOVA two-tailed Dunnett’s approach for multiple comparisons relative to the H^cwt^ control (*p < 0.05; **p < 0.01; ***p < 0.001)

Each effector line was crossed with the *sd*-Gal4; *RBPJ*^*wt*^ driver line to induce the overexpression of the respective H* mutant protein in the salivary glands of *RBPJ*^*wt*^ larvae. Staining of the salivary glands then revealed the subcellular distribution of H* and RBPJ^wt^ protein, respectively (Fig. 2a). As shown earlier [29], H^*NLS3^ protein was mostly cytoplasmic, whereas all other H* variants were detected in both, cytosolic and nuclear compartment. Notably, H^*NES^ appeared more strongly enriched in the nucleus than H^cwt^ and H^*NLS3*NES^ protein (Fig. 2a). As predicted from the subcellular localization of Su(H) [29], RBPJ^wt^ protein was cytosolic when H^*NLS3^ protein was overexpressed, and detected in the nuclear compartment as well in the presence of any other H* protein variant (Fig. 2a). To confirm the visual impression, we quantified the staining intensities of confocal micrographs on eight specimen each for every genotype, comprising a minimum of 160 nuclei. Composite Z-stacks crossing the entire gland were segmented into nuclei and cytoplasm, and mean grey values were recorded. The results confirm that murine RBPJ^wt^ protein is shuttled by H protein the same way as is Su(H) protein (Fig. 2b). Overexpression of the wild type protein isoform H^cwt^ caused strong accumulation of RBPJ^wt^ protein in the nucleus, and even stronger, when H^*NES^ was overexpressed. In contrast, overexpression of H^*NLS3^ resulted in the retention of RBPJ^wt^ in the cytoplasm, whereas that of H^*NLS3*NES^ allowed RBPJ^wt^ protein to re-enter the nucleus (Fig. 2b). Briefly, we observed a nucleo-cytosolic shuttling of RBPJ^wt^ protein, which strictly followed H protein distribution in the salivary glands of *Drosophila* larvae.

RBPJ^wt^ protein accumulated significantly stronger in nuclei upon the overexpression of H^*NES^ (Fig. 2) which is defective in the nuclear export signal [29]. Evidently, RBPJ^wt^ is subjected to nuclear export by wild type H protein similar to Su(H). The importance of nuclear export of CSL-H has not been elucidated yet. We know, however, that the H-NES is relevant for fly survival, as in its absence, only a fraction of the animals developed to adulthood [29]. In mouse cells, the tubulin-binding protein RITA induced nuclear export of RBPJ, thereby downregulating Notch-mediated transcription [33]. The more important roles of RITA, however, lie in the regulation of microtubule dynamics during mitosis and cell motility [34, 35]. Albeit its high conservation in the animal kingdom, RITA has no fly homologue. Accordingly, despite binding to Su(H) and tubulin, human RITA has no biological effect on the subcellular distribution or the stability of Su(H) protein in the fly [36]. In contrast to mammalian cells, sequestration of Su(H) by a tubulin-tether in the cytosolic compartment does not occur [36]. Nevertheless, regulation of nuclear availability of CSL proteins appears an important layer of regulation during the transduction of Notch signals in vertebrates and equally in invertebrates.

## Conclusion

Nucleo-cytoplasmic shuttling of Su(H) as a means of regulating Notch signaling activity in the fly has been already shown. Here we demonstrate that murine RBPJ is subjected to a likewise dynamic nucleo-cytoplasmic shuttling by H protein in vivo in *Drosophila* tissue. These data support the hypothesis that nuclear availability of mammalian CSL proteins is restricted by their cofactors, on which they depend for nuclear import. Moreover, murine RBPJ protein is also subjected to nuclear export by H protein. Overall, our data demonstrate the requirement of corepressors for CLS nuclear translocation, emphasizing the additional layer of regulation at the level of nuclear availability.

## Methods

The genome engineered fly stock *RBPJ*^*wt*^ / CyO-GFP contains murine RBPJ cDNA (isoform 1; the N-terminal 128 codons are derived from Su(H) fused at Val-codon 81 to RBPJ) in place of wild type Su(H) [30]. The stock was combined with *sd*-Gal4 (BL8609) to generate a driver line, and with either UAS-*H*^*cwt*^, UAS-H^*NLS3^, UAS-H^*NES^ or UAS-H^*NLS3*NES^ [29] to generate an effector line, by standard genetic crosses as outlined in Fig. 1. To this end, we made use of the dominant markers *sna*^*Sco*^ (BL9325) and *L*^*2*^ (BL319) [37], and a doubly balanced *cycG*^*HR7*^ allele [38], to be able to follow unambiguously every chromosome through all generations. Driver and effector lines were crossed, and offspring reared at 25 °C to eventually analyse the salivary glands at third instar larval stage. The homozygous *RBPJ*^*wt*^ animals were recognized by the lack of GFP, otherwise marking the heterozygous siblings due to the CyO-GFP (BL9325) marker. A Leica MZ FLIII UV stereo-microscope (Leica, Wetzlar, Germany) illuminated with CoolLED pE-300^white^ (AHF, Tübingen, Germany) was used for the purpose of selecting the larvae.

Respective UAS-constructs were expressed in the developing salivary glands using *sd*-Gal4. To visualize H and RBPJ protein expression, immuno-cytochemistry on third instar larval salivary glands was performed as outlined before, with a 20 min fixation with 4% paraformaldehyde [29]. As primary antibodies, we used guinea pig anti-Hairless A (1:500) [27] and rabbit anti-RBPSUH (1:200) (D10A4; Cell Signaling Technology, Cambridge, UK). Goat secondary antibodies (1:250), coupled to FITC or Cy3 were obtained from Jackson Immuno-Research (Dianova, Hamburg, Germany). Fluorescently labelled tissue was mounted in Vectashield (Vector labs, Eching, Germany). Pictures were taken with a Zeiss Axioskop (Carl Zeiss, Jena, Germany), coupled to a BioRad MRC1024 confocal microscope (Carl Zeiss, Jena, Germany; O.S.T.I. microscopy, Milano, Italy) using LaserSharp 2000™ software. The presented figures were created using *ImageJ*, *PhotoPaint* and *CorelDraw* software.

Quantification of H and Su(H) protein in salivary glands overexpressing the specific H* nuclear localization mutant was performed based on confocal micrographs using *Image J* software. Z-stacks crossing the entire glands with 1 μm increments were used for maximum projection. Composite images were segmented into nuclei and cytoplasm. Nuclei were defined as region of interest, and subtracted from the outline of the whole gland, defining the cytoplasm. Mean grey values of nuclei and corresponding cytoplasm of the entire gland were recorded [29]. Eight glands each with a total of at least 160 nuclei were analyzed. Statistical significance was determined by ANOVA two-tailed Dunnett’s approach for multiple comparisons.

## Data Availability

All data generated or analyzed during this study are included in this published article.

## Abbreviations

CBF1: C promoter binding factor 1 (mammalian)
CSL: *C*BF1/RBPJ, *S*u(H), *L*ag-1 (acronym)
Gal4: *gal*actose responsive transcription factor (from *S. cerevisiae)*
GFP: Green fluorescent protein (from *A. Victoria)*
H: Hairless (from *D. melanogaster)*
HDAC1: Histone deacetylase 1 (mammalian)
Lag1: Abnormal cell *lin*eage 12 (lin-12) and abnormal *g*erm *l*ine *p*roliferation phenotype-a (glp-1) (from *C. elegans)*
Mam: Mastermind from *D. melanogaster*; corresponds to mammalian MamL (mastermind like transcriptional coactivator)
MINT: Msx2-interacting nuclear target (mammalian)
NCOR2: Nuclear receptor corepressor 2 (mammalian)
NES: Nuclear export signal
NICD: Notch intracellular domain
NLS: Nuclear localization signal
RAM: RBP-Jkappa-associated module (part of NICD)
RBPJ: Recombination signal binding protein for immunoglobulin kappa J region (mammalian)
RITA: RBPJ interacting and tubulin associated, also named ZNF331 (mammalian)
SHARP: SMRT/HDAC1-associated repressor protein (mammalian)
SMRT (or NCOR2): Silencing mediator for retinoid and thyroid-hormone receptors (mammalian)
Su(H): Suppressor of Hairless from *D. melanogaster*
UAS: Upstream activating sequences (Gal4 target sequence)
ZNF331: Zinc finger protein 331 (mammalian)
